# Perception of Time in Music in Patients with Parkinson's Disease–The Processing of Musical Syntax Compensates for Rhythmic Deficits

**DOI:** 10.3389/fnins.2017.00068

**Published:** 2017-02-23

**Authors:** Daniel Bellinger, Eckart Altenmüller, Jens Volkmann

**Affiliations:** ^1^Department of Neurology, University Hospital WürzburgWürzburg, Germany; ^2^Institute of Music Physiology and Musicians' Medicine, University of Music, Drama and MediaHannover, Germany

**Keywords:** Parkinson disease, psychophysics, time perception, rhythm perception, musical syntax, just noticeable difference (JND)

## Abstract

**Objective:** Perception of time as well as rhythm in musical structures rely on complex brain mechanisms and require an extended network of multiple neural sources. They are therefore sensitive to impairment. Several psychophysical studies have shown that patients with Parkinson's disease (PD) have deficits in perceiving time and rhythms due to a malfunction of the basal ganglia (BG) network.

**Method:** In this study we investigated the time perception of PD patients during music perception by assessing their just noticeable difference (JND) in the time perception of a complex musical *Gestalt*. We applied a temporal discrimination task using a short melody with a clear beat-based rhythm. Among the subjects, 26 patients under L-Dopa administration and 21 age-matched controls had to detect an artificially delayed time interval in the range between 80 and 300 ms in the middle of the musical period. We analyzed the data by (a) calculating the detection threshold directly, (b) by extrapolating the JNDs, (c) relating it to musical expertise.

**Results:** Patients differed from controls in the detection of time-intervals between 220 and 300 ms (^*^p = 0.0200, *n* = 47). Furthermore, this deficit depended on the severity of the disease (^*^p = 0.0452; *n* = 47). Surprisingly, PD patients did not show any deficit of their JND compared to healthy controls, although the results showed a trend (^*^p = 0.0565, *n* = 40). Furthermore, no significant difference of the JND was found according to the severity of the disease. Additionally, musically trained persons seemed to have lower thresholds in detecting deviations in time and syntactic structures of music (^*^p = 0.0343, *n* = 39).

**Conclusion:** As an explanation of these results, we would like to propose the hypothesis of a *time-syntax-congruency in music perception* suggesting that processing of time and rhythm is a Gestalt process and that cortical areas involved in processing of musical syntax may compensate for impaired BG circuits that are responsible for time processing and rhythm perception. This mechanism may emerge more strongly as the deficits in time processing and rhythm perception progress. Furthermore, we presume that top-down-bottom-up-processes interfere additionally and interact in this context of compensation.

## Introduction

Parkinson's disease (PD) is a movement disorder of neurodegenerative nature affecting the basal ganglia (BG), particularly the nigrostriatal system. Traditionally, symptoms in the motor domain dominate the clinical picture and their features play a crucial role in the process of PD diagnosis (Hughes et al., 1992). Nevertheless, non-motor symptoms of PD should not be neglected when considering their substantial impact on the patients' daily lives (Todorova et al., 2014). These symptoms, such as chronic pain, depression, anxiety, cognitive dysfunction or autonomic disorders often have to be treated separately and turn out to be an additional challenge in long-term therapy (Chaudhuri and Schapira, 2009). In recent years, PD research has also focused on sensory deficits associated with the disease. Notably, the perception of time and rhythm has attracted considerable attention. Already in 1997, Rammsayer described a significant impairment in temporal discrimination in PD patients using intervals in the range of milliseconds (Rammsayer and Classen, 1997). Following studies on temporal processing emphasized the importance of the BG network in “timing operations” (Harrington et al., 1998a). Furthermore, in a review based on several studies on time perception, Lucas et al. came to the conclusion that “*due to a BG dysfunction the sensorimotor integration in the posterior parietal cortex is impaired*” in patients with PD (Lucas et al., 2013). Interestingly, Guehl et al. who conducted psychophysical tests on 18 PD patients with deep brain stimulation in the STN concluded that the deficits in temporal discrimination reflect an “*impairment in memory and/or attention rather than in the perception of time per se*” (Guehl et al., 2008, p. 1).

In a previous study with 15 PD patients, it was reported that patients were less able to perceive a beat-based rhythm compared to a complex rhythm (Grahn and Brett, 2009). This is remarkable, as one would expect that a given meter facilitates the discrimination of a time interval and that the perception of a complex rhythm is compromised. Instead, the authors conclude that only the perception of a beat-based rhythm is impaired. fMRI studies confirmed the crucial role of the putamen, pallidum and Ncl. caudatus in temporal processing and beat perception (Nenadic et al., 2003). These structures are considered to contain networks involved in a beat-based rhythm response (Grahn, 2009; Shih et al., 2009; Benoit et al., 2014). Interestingly, previous studies and recent findings about rhythm perception in PD have also demonstrated the clinical value in using rhythmic stimuli or music especially for rhythmic cueing in gait-training (Enzensberger and Fischer, 1996; Lim et al., 2005; Nombela et al., 2013), for example, the *Rhythmic auditory stimulation* (Thaut et al., 1996) or the *Ronnie Gardiner Rhythm and Music (RGRM*™*) Method* (Pohl et al., 2013). In addition, research into PD patients with deep brain stimulation of the STN show an improvement in the perception of time (Koch et al., 2004). These findings lead to the question whether PD impairs the perception of rhythm when patients are presented with music. Therefore, we developed a psychophysical experiment in which we investigated the detection thresholds of patients. Our interest was especially the just noticeable difference (JND) for temporal deviations in a melody. Patients and controls heard a short melodic sequence with an artificially manipulated time delay (interval) in the sub-second range (milliseconds) which had to be detected.

Our hypotheses were (1) that PD patients are less able to perceive a time-interval (musical or artificial pause) within a melody which implicates a clear, simple metric rhythm and (2) that in contrast to healthy controls, PD patients show altered auditory detection thresholds reflected in the (JND). This affects the processing of temporal structures—including rhythmic structures as in music—as well as the processing of musical syntax. Furthermore, we hypothesized (3) that this sensory discrimination ability would be impaired in PD patients in advanced stages of the disease.

As musical syntax we understand musical structures which can, – according to rules –, be assigned to higher-ranking, hierarchical principles. Such an establishing of functional relations between musical elements can be understood in analogy to linguistic syntax. Interestingly, the processing of musical syntax seems to be closely related to the processing of linguistic syntax, both in terms of functional organization and underlying of brain mechanisms (e.g., Maess et al., 2001; Patel, 2008). An intense interaction of both domains has been described in several studies (Fitzroy and Sanders, 2012; Koelsch, 2013, p. 138; Donnay et al., 2014). However, studies on the processing of musical syntax in the temporal domain are rare since research focused on the syntactic processing of harmony (for a detailed review see Koelsch, 2011). Surprisingly, even non-musicians show an implicit knowledge about the rules of musical syntax (Koelsch, 2005; Brattico et al., 2006). Related to the question of musical syntax in temporal structures is research into phrase structures in music, as undertaken by Knösche et al. (2005). They investigated the perception of phrase structures in music emphasizing particularly phrase boundaries, which facilitate phrase processing in the human brain and which can also be understood as analogy to phrase boundaries in language (Knösche et al., 2005): Their neural correlate, the closure positive shift, also shows resemblance to neural correlates of prosodic phrase boundaries. However, regarding the designs of these studies on phrase structures, the processing of musical syntax has to be distinguished from processing of phrase boundaries because in contrast to multi-leveled hierarchies of musical syntax, phrase structuring only describes “*one level in a complex hierarchy*” as to be seen below (Stoffer, 1985; Knösche et al., 2005, p. 1).

## Materials and stimulus

### Participants and assessments

Participants in the study included 26 PD patients (16 male, 10 female) and 21 controls (9 male, 12 female) between the ages of 45 and 80 years (patients mean 64.6; SD 7.9; probands mean 58.4; SD 9.1). The experiment was approved by the ethics institutional review board of the university hospital. The confidentiality of data was ensured. Foremost, all participants underwent a neuropsychological test, the *Parkinson Neuropsychometric Dementia Assessment, PANDA* (Kalbe et al., 2008) and an audiometry before the psychophysical experiment was conducted. Patients with dementia or in the last stage of the disease (Hoehn & Yahr 5) were excluded. The MDS-UPRRS (motoric part III) was carried out with PD patients to quantify their current motoric deficit. In addition, their dopaminergic medication was recorded in the Levodopa—equivalent dosages; two patients were under deep brain stimulation. The musical listening biography and the musical expertise of the participants were also important points of interest. We assessed musical activity with a questionnaire referring to the subjects' musical biographies based on findings on musical expertise by Müllensiefen et al. (2014). It contained six questions concerning the subjects' musical experiences (see Table 1 of the Supplementary Material). Subsequently, we categorized the individual music comprehension of the participants into three different categories (1–4 points: low musical comprehension, 5–8 points: average musical comprehension, 9–12 points: high musical comprehension).

### Psychophysical experiment

#### Stimulus and its gestalt principles

**Figure 1:**
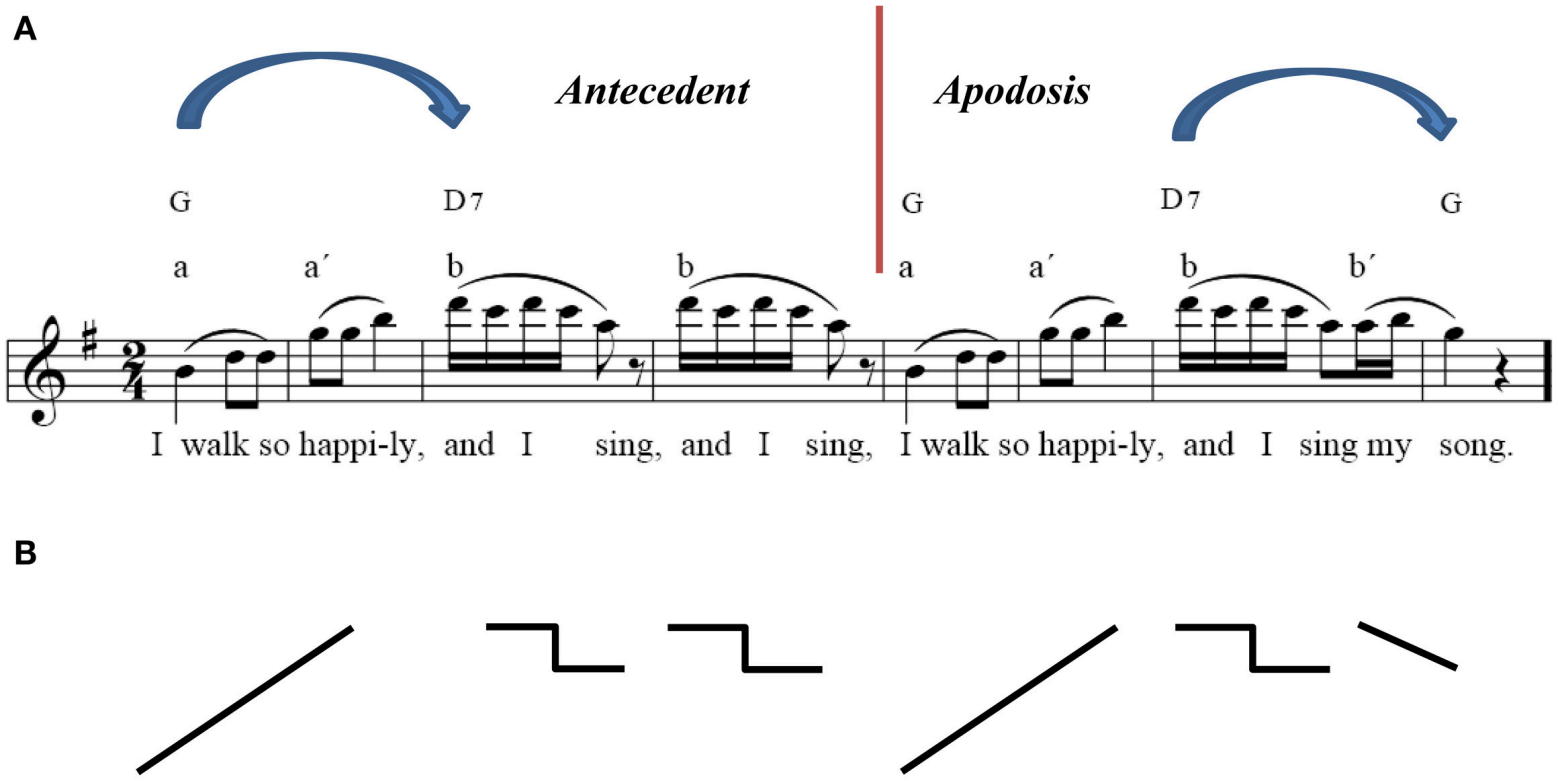
**Music syntactical organization of the stimulus and its extraction by means of the Gestalt principles: closeness, nearness, similarity, symmetry, good continuation**. The similarities to linguistic syntax processing and the perception of melody are elucidated in detail by Patel (2003), p.326–334; 2008. For further explanations see text.

It was essential to find an appropriate melody with a clear beat-based rhythm, which was easy to identify at the first listening and which required no formal musical training to identify. The main theme, the first eight bars of Beethoven's Capriccio op. 129, was chosen because of its simple melodic structure, its potential for rhythmic entrainment, and its musical characteristics, which are optimally concordant with psychological Gestalt principles (see Figure 1; The similarities to linguistic syntax processing and the perception of melody are elucidated in detail by Patel, 2003, p.326–334; 2008). To ensure a pleasant piano sound (the stimulus had to be heard about 100 times), the melody was played by a pianist on a Clavinova and recorded by a common sound editor (*Goldwave*, Kelly® Media AG, 2001, Version 4.22). The clear line of the melody containing all tones of the G major triad, the repetition of the first phrase, the rhythmic emphasis on accentuated counts and, therefore, the implication of a clear musical meter are those core characteristics corresponding to the Gestalt principles in music perception. These were crucial for memorization, integration in time and anticipation of the melody, which the participants had to achieve. Despite the “simple” harmony and rhythm, the musical sequence must be considered as a complex harmonic figure requiring holistic perceptive processes including rule extraction, anticipation and memorization of the stimulus within the brain. As a critical manipulation of the melody, an artificial interval for the duration of 20 ms up to 700 ms was inserted after the first four bars, following the second eighth note pause, which equated to a duration of 380 ms while being played with 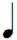 = 100 bpm. At this position we find a “musically natural” interval which evokes an expectancy for something new within the melody. Therefore, we decided to manipulate this anticipation by inserting additional time-intervals which were meant to change the musical structure only in a subtle way without generating any unnatural, obvious break or arousal.

#### Procedure

**Figure 2:**
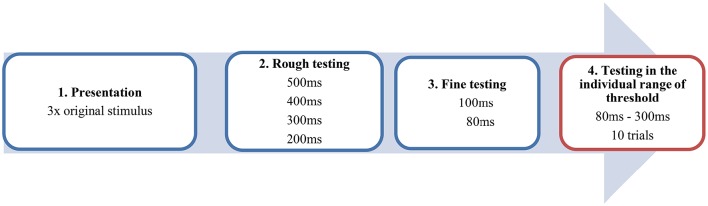
**Example of the procedure in four steps**. The blue windows contain the steps of the pretest. The red window reflects the testing within the individual range of threshold conducted by a pseudorandomized procedure.

All patients were tested under the L-dopa administration in the ON MED Status. The musical sequence in its original, unaltered version was presented to the participants three times binaurally by a headphone and had to be memorized. In the following task, patients and controls had to decide whether the time interval between the antecedent and apodosis was correct or manipulated. The experiment was conducted in four steps (see Figure 2). After the presentation of the original stimulus, we started with the altered stimulus including a large artificial interval of 500 ms, which was recognized by most of the subjects. Then we successively tested from the higher to the lower range of perception in 100 ms interval-steps in order to approach the individual range of threshold (red window). Of course, this differed individually but reflected mostly a time window between 80 and 300 ms. Now, using 20 ms interval steps it was possible to test in the ideal psychophysical range of perception of the participants. This testing window was conducted in 10 trials including 11 to 12 different time-intervals. The subject confirmed by oral response (“right/false” or “no pause/pause”) whether the original stimulus or the altered stimulus was heard. Because of a pseudorandomized procedure, subjects did not know which stimulus was presented. It is important to emphasize that in the testing window of the individual range of threshold (see Figure 2, red box), the subjects only heard altered versions of the stimulus which they thought to be the original. Only the data of this part of the experiment were included in the final analysis.

## Results

**Figure 3:**
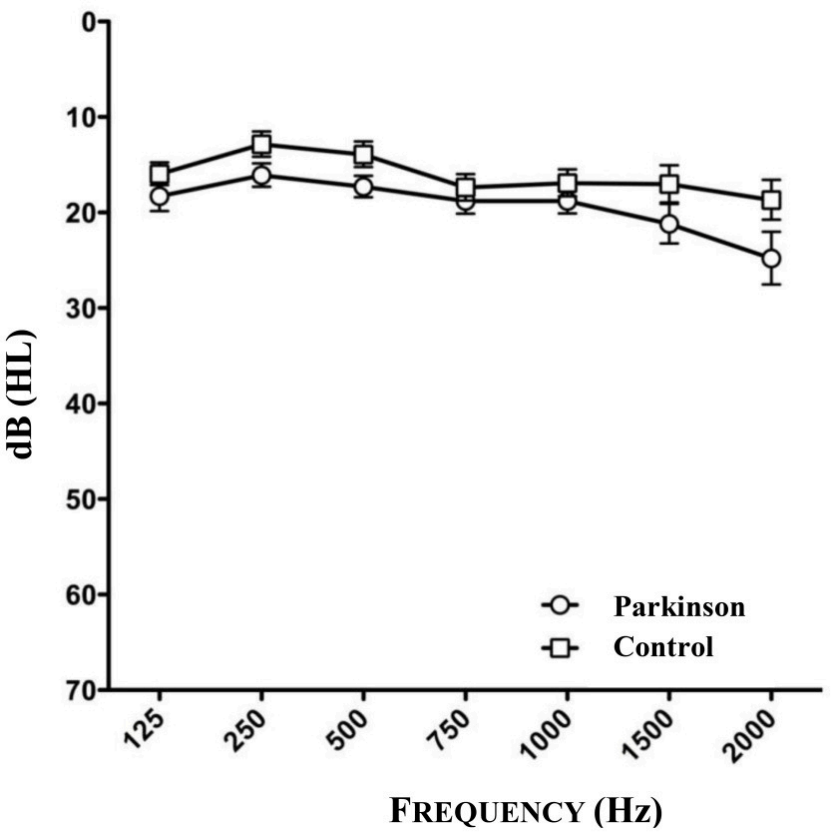
**Results of audiometry of patients and healthy controls (*n* = 46)**. One patient could not be tested because of the inaccessibility to audiometry.

The audiometry of patients and controls showed the age related physiological hearing loss to be between 10 and 25 dB for the frequencies between 250 and 1,500 Hz (see Figure 3, *n* = 46). Epidemiological and clinical characteristics of patients and healthy controls can be seen in Tables 1–3.

**Table 1:** **Age and sex of patient and healthy controls**.

		**Group (*n* = 47)**			
		**Patients**		**Healthy controls**	
		**Male**	**Female**	**Male**	**Female**
**Age**	*N*	16	10	9	12
	Mean value	65.9	62.4	60.4	57.2
	SD	7.9	8.2	10.8	8.3

**Table 2:** **Results of the PANDA Test**.

		**Group (*n* = 47)**	
		**Patients**	**Healthy controls**
PANDA Cognition (1–30 points)	*N*	25	21
	Mean value	23.1	24.1
	SD	4.6	4.5
PANDA Mood (1–4 points)	Mean value	1.2	1.3
	SD	0.9	1.2

*The PANDA cognition test is a global cognitive screening tool for Parkinson dementia with a range of 1–30 points: Scores above 18 points indicate normal cognitive function, 15–17 points mild cognitive dysfunction, <14 points a probable dementia. Depression is screened by the PANDA Mood subtest with a score >4 indicating likely depression*.

**Table 3:** **Clinical characteristics of patients**.

		**Patients (*n* = 26)**
Hoehn & Yahr (1–5)	Mean value	4.3
	SD	2.1
Duration of disease (years)	Mean value	10.1
	SD	5.7
UPDRS III (ON MED)^*^	Mean value	29
	SD	16.8
Freezing (1–4 points)	Mean value	0.8
	SD	1.5
LEDD (mg)	Mean value	1045.5
	SD	871.8

* *NB: 0–137 possible points. Please note that the UPDRS III reflects the total severity of all Parkinsonian motor symptoms with higher values indicating more severe motor deficits. ON MED describes the patients status under current dopaminergic medication*.

### Approach to experimental data

Because of the small sample (*n* = 47) and based on the fact that a normal distribution could not be assumed between patients and controls we applied non-parametric test methods, such as the Wilcoxon-Mann-Whitney Test (*U*-Test) for two unconnected samples and the Kruskal Wallis Test for more than two groups. To countervail the alpha error we conducted paired comparisons by Steel Dwass. Correlation calculations were realized by Spearman (ρ).

Statistical significance was presumed at a α-level of ^*^p < 0.05. The individual testing of the subjects mostly ranked in the interval range of 80 and 300 ms. The data were analyzed in two different ways. First, the data of all subjects were considered generally and included the results of all correctly identified intervals (*n* = 47).

In contrast, the JND of every subject was detected by means of linear regression and extrapolation, which depended on the critical value of the correlation coefficient. Therefore, the results of seven subjects had to be rejected, which meant that we had to exclude the data of the two DBS patients. After correction of the data, the JND results of 40 subjects were available for the evaluation (20 patients, 20 probands).

### General comparison of experimental data

**Figure 4:**
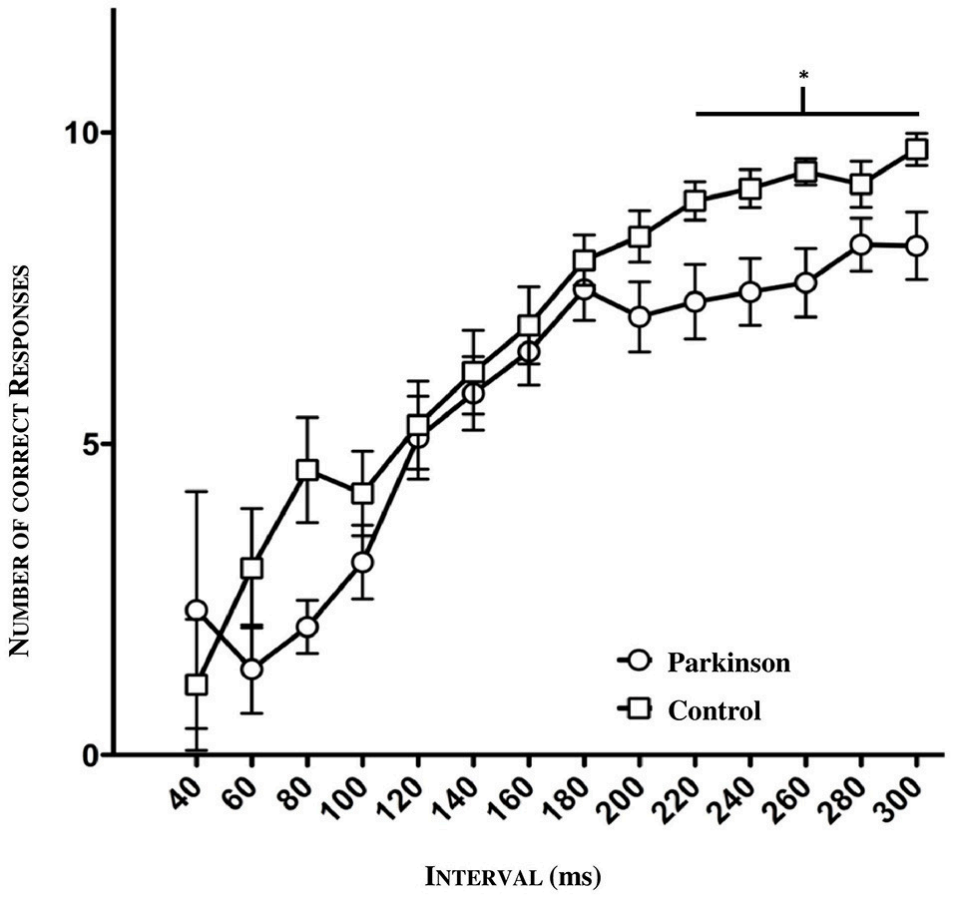
**Psychometric function of patients and controls (*n* = 47)**. Significance in the higher time window between 220 ms and 300 ms (*p = 0.0200).

The psychometric function as seen below (Figure 4) reflects the number of all correct responses plotted against the mean value and its standard deviation (SD) in the tested interval. A clear discrepancy between patients and controls is apparent, especially concerning the interval range between 40 and 80 ms as well as the range between 200 and 300 ms. In the lower range (40–100 ms), the groups showed enormous fluctuations of standard deviation due to the fact that just a few subjects were tested in this window. Hence, the large standard deviation can be explained. In order to detect a possible significance in this lower and higher time window between patients and controls, we divided the intervals into three sections (lower section: 60–100 ms, middle section: 120–200 ms; higher section: 220–300 ms). In the lower and middle sections, no significant difference was found. However, in the higher time window, a significance was detected between 220 and 300 ms (^*^p = 0.0200, *n* = 47, see Figure 4).

**Figure 5:**
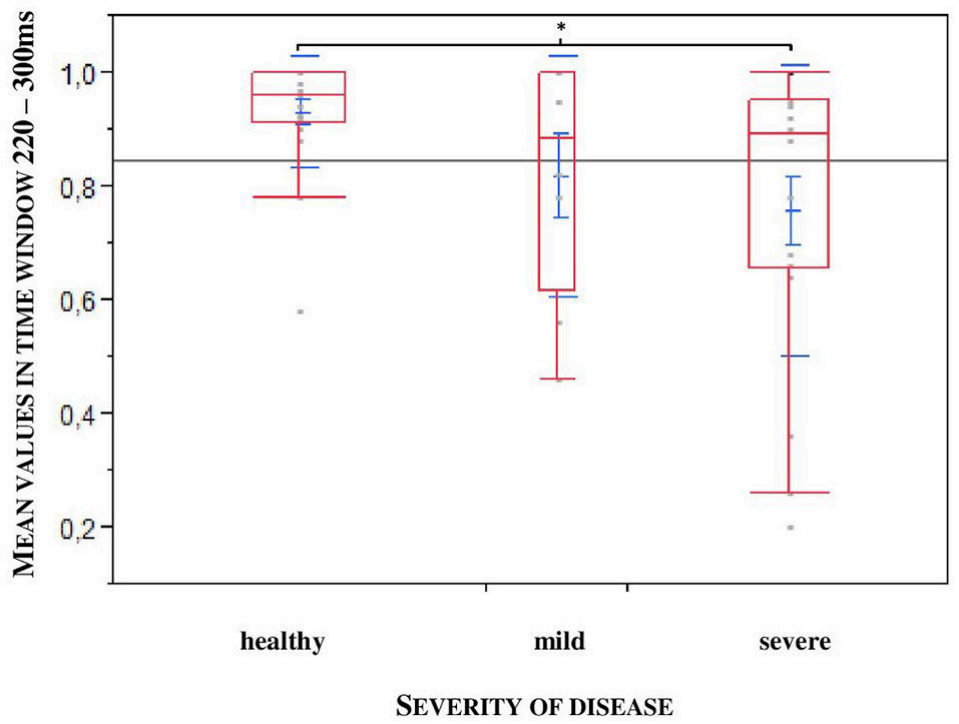
**Larger time window depending on the severity of the disease (mild: H&Y stage 1–2,5; severe: H&Y stage 3–4)**. Significance especially between healthy controls and severe affected patients. (*p = 0.0317).

Furthermore, to uncover a potential difference according to the stadium of the disease we divided the patients into two groups: mild: H&Y stage 1–2.5; severe: H&Y stage 3–4. We found out that exactly in the larger time window, a statistical significance could be observed relating to the severity of the disease (^*^p = 0.0452; *n* = 47), especially between healthy controls and severely affected patients (*p = 0.0317, *n* = 47, see Figure 5).

### Specific considerations: just noticeable difference

**Figure 6:**
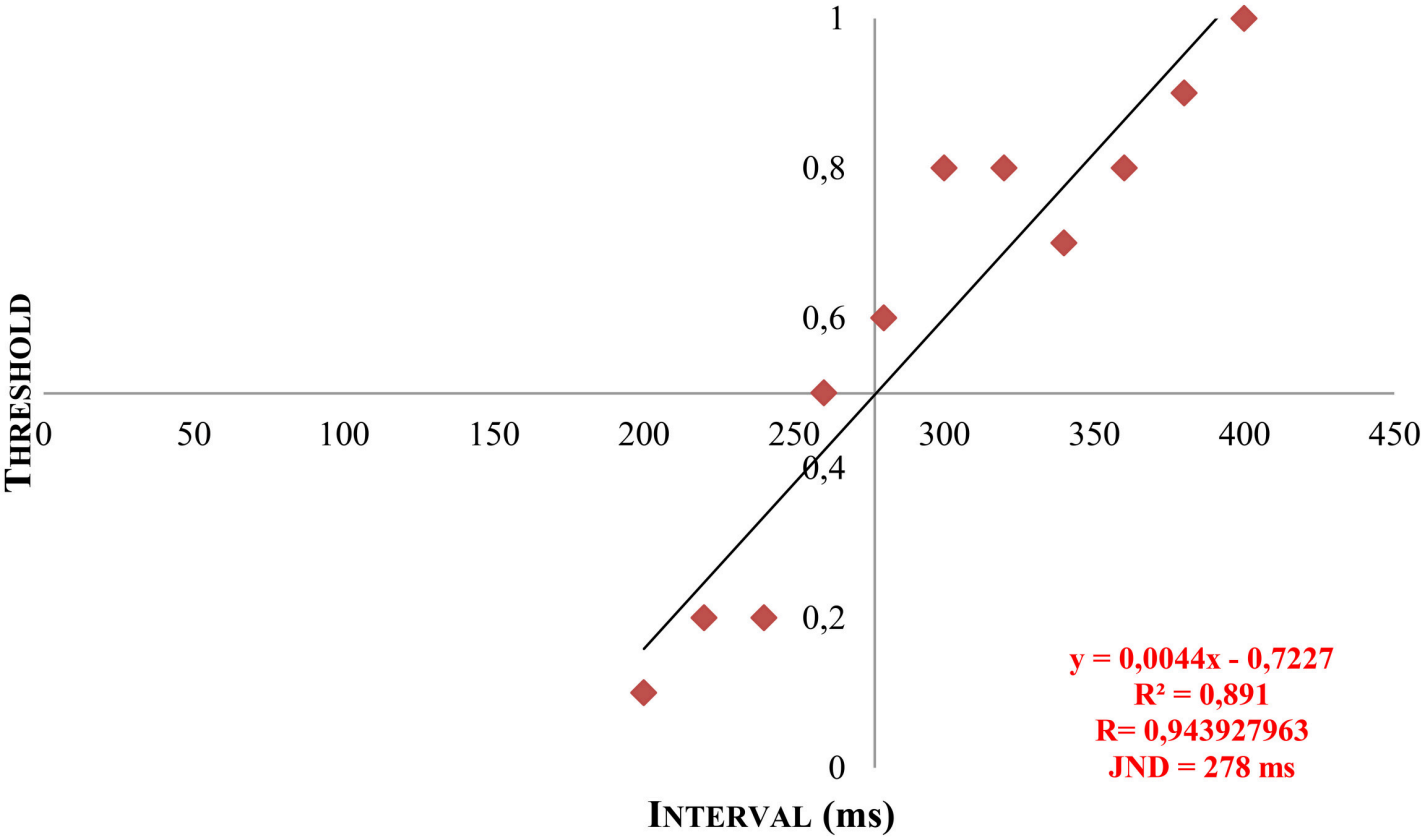
**Example of a patient's JND by means of linear regression and extrapolation**. *Y* = calculated gradient of the regression line based on the measured data. *R*, correlation coefficient. *R*^2^, coefficient of determination. *x*, calculated JND by extrapolation at *y* = 0.5.

With the psychophysical testing, a numeric matrix of data was established for every participant and could be converted into a psychometric function by means of linear regression and extrapolation. Thus, the individual discrimination threshold (JND) was generated and illustrated graphically (see Figure 6).

#### JND and PD

The comparison between patients in ON MED and the controls showed a different picture: No difference between the groups was found, although the results came very close to significance (^*^p = 0.0565, *n* = 40). Furthermore, no significant difference was found related to the severity of the disease.

#### JND and musical comprehension

**Figure 7:**
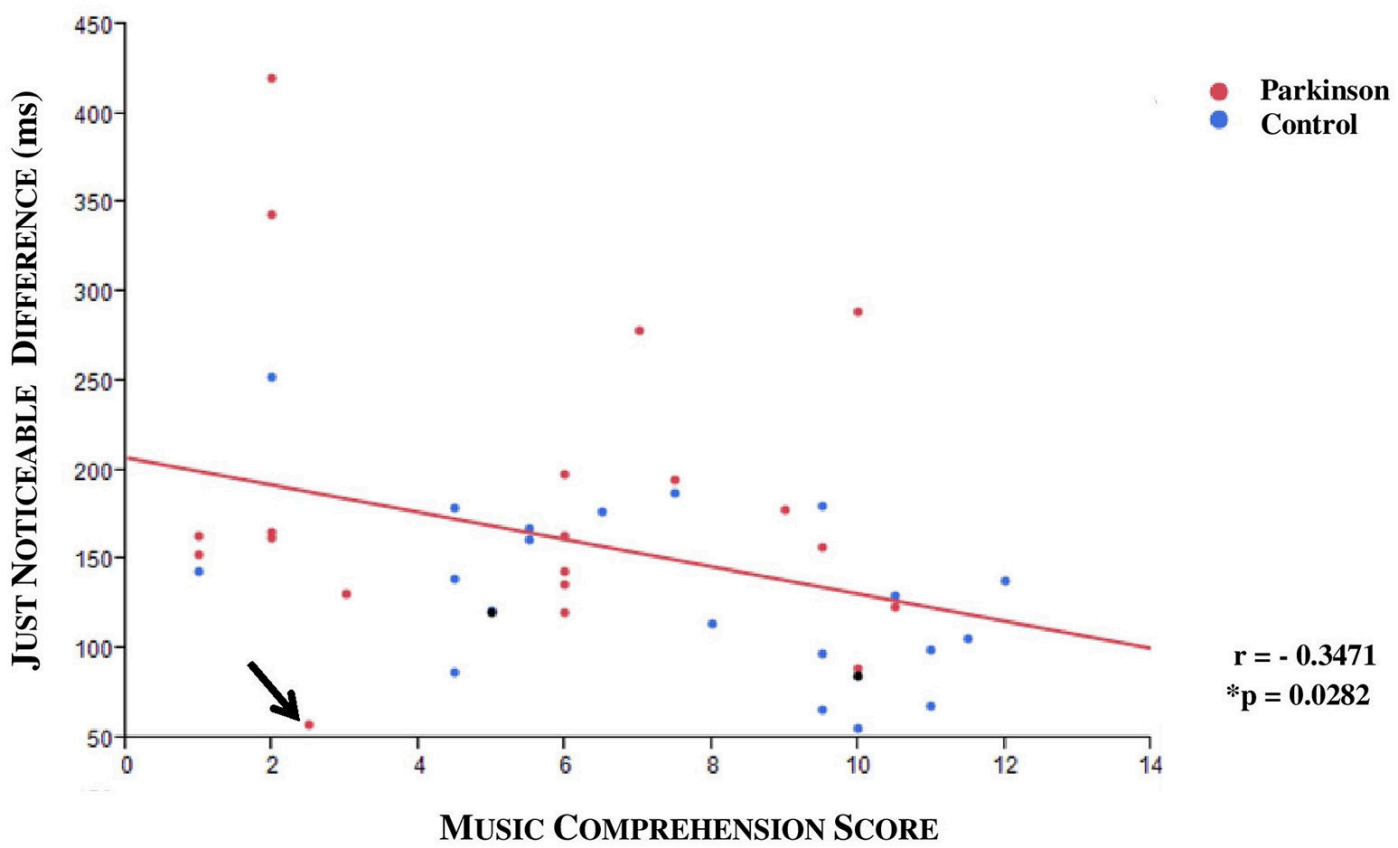
**Scatterplot with regression line (red): JND and musical comprehension**. An outlier was identified (arrow). A negative correlation was displayed (*r* = −0.3471; ^*^p = 0.0282, *n* = 40; both DBS patients who were excluded from the analysis are also shown in the graphic as black points).

**Figure 8:**
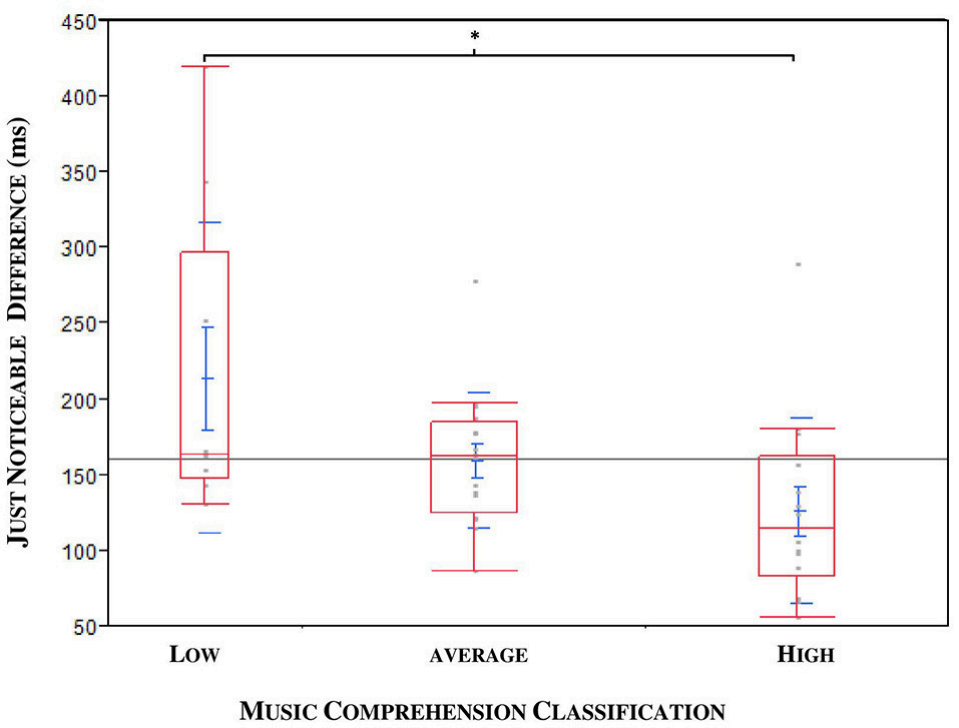
**JND compared to the musical expertise of patients (ON MED) and probands in three categories: DBS patients and outlier excluded**. Significance especially between the categories “low” and “high” (*p = 0.0343).

A medium negative correlation between JND and musical expertise was found (ρ = −0.3471, ^*^p = 0.0282; *n* = 40; see Figure 7). The musical comprehension between patients and controls did not differ for the 40 subjects in the JND analysis (^*^p = 0.0899, *n* = 40), although the evaluation of all subjects came close to significance (^*^p = 0.052, *n* = 47). An outlier was identified; this patient had a JND of 58 ms but only achieved a low score in music comprehension although he should have been classified as a subject with high musical comprehension. Under exclusion of the outlier we could observe a significance by means of the Kruskal Wallis Test (*p = 0.0221; *n* = 39). To specify the category, we carried out a Steel-Dwass pair comparison which excluded the outlier. A significant difference especially between the group with higher and lower musical expertise could be ascertained (^*^p = 0.0343, *n* = 39, without outlier, see Figure 8).

## Discussion

In the last years, the focus on non-motor symptoms in PD has resulted in several psychophysical experiments concerning visual, tactile and auditory perceptive thresholds. In the present study we required the integration of time and rhythm perception, but also of music perception, especially the extraction of musical syntax. We therefore probed several cognitive processes during music perception. Specifically, we tested the integration of pitch and time information of melody, meter, rhythm and Gestalt perception. Patients and controls had to detect minimal changes within the temporal structure of the melody which affected musical syntax in a significant way. The induction of a clear rhythm was thought to facilitate the perceptive discrimination of the stimulus from the melody, respectively. Possible perceptive deficits in temporal and melodic structures in patients with PD would thus be detected.

The results in the longer time window (220–300 ms) were significant and indicated–in keeping with the literature–that PD patients are impaired in their temporal discrimination ability, especially in the integration of a longer time interval >600 ms (380 ms musical pause + 220 ms artificial interval). Deficits in time processing have already been described in previous studies (Artieda et al., 1992; Rammsayer and Classen, 1997). Rammsayer and Artieda argue that this impairment in patients with PD is due to the pathologically affected pacemaker – namely the BG – necessary for the perception and estimation of time. Artieda et al. report a significant correlation with the stage of disease. On the other hand, Guehl et al. report deficits in PD patients only in two of six duration discrimination tests, which are similar to Rammsayers' tests (Guehl et al., 2008). The authors contradict the view of Rammsayer and Artieda and explain the results as a dysfunction of the auditory cortex and an impairment rather of attention and memory in PD patients. Deficits in attention and working memory have also been emphasized by other authors (Harrington et al., 1998b; Koch et al., 2008; Poryazova et al., 2013). Interestingly, Koch et al. report of no impairment in PD patients in the time reproduction in the supra-second range >2 s (Koch et al., 2008). Ivry et al. emphasize the crucial role of the cerebellum in time processing (Ivry, 1996; Ivry and Spencer, 2004). They suggest that the BG process sensory-motor events in longer time intervals than the cerebellum which processes shorter intervals.

Taken together, the significant results of deficits in the longer time estimation range should be interpreted carefully (see Figure 4). Although these results suggest a general time deficit in PD patients, the deductive conclusion fails, however, because the results of the JND analysis show no difference between patients and controls explicitly. This supports the idea that perception in PD patients, especially the just noticable difference, which reflects the more individual ability of time perception, is not necessarily impaired and could be unaffected. Indeed, PD compromises auditory temporal processing, even if this is not clinically evident. Despite the results of our study, the comparability to other psychophysical studies is limited because of their different methodical approaches.

### Proposal of a compensation model: processing of musical syntax as a contextual factor

Extending the work of Grahn, who primarily conducted psychophysical experiments based on rhythm perception in PD patients, we argue that the processing of musical syntax plays an important role in the processing of temporal structures in general when time is considered explicitly as a parameter in a musical context of perception. Grahn and Brett report a deficit in PD patients in detecting a metric simple rhythm (Grahn and Brett, 2009). The clear beat-based rhythm structure of the stimulus in our experiment was meant to help the subjects perceive it and could be considered as a resource for detecting the manipulated time interval within the melody. Hence, we should have expected the results of the JND between patients and controls to be significant, because the “clear beat-based rhythm” could aid the healthy controls, but not the PD patients, who suffer an impairment of beat-based rhythm perception. Therefore, with this explanation, the emphasis is laid on the processing of musical syntax as a contextual factor. This could actually be unimpaired in PD patients, as mentioned above (or at least be reconstituted under medication) since the processing of musical syntax also involves other multineuronal pathways and different anatomical structures: e.g., Brodmann Area 6, 22 and 44, elucidated in the works of Koelsch recently (Koelsch, 2011, 2013, p. 90). Besides, other neural correlates of time processing, such as the cerebellum, should not be neglected (Ivry and Spencer, 2004). It should be mentioned, that an alternative explanation could be the phrase structuring as mentioned above. Indeed, intact processing of phrase structures and explicitly of phrase boundaries which is electrophysiologically reflected by the closure positive shift (CPS) could also explain the unimpaired sensory ability (JND) of PD patients (Knösche et al., 2005). In this context, Knösche emphasizes the important fact, that the CPS reflects working memory and attention needed within this process. However, the deficits of PD-patients in time estimation in the time window of 80 ms and between 200 and 260 ms points toward a more complex pattern of pathological alterations which cannot be explained sufficiently by a model based on phrase structuring.

As a possible explanation, we propose the *hypothesis of time-syntax-congruency in music perception*: Temporal, rhythmic and music syntactic structures cannot be considered and examined separately from each other, but rather build up a perceptive unity. This unity is the basic principle of music, yet it has to be considered more precisely and sophisticatedly in an experimental and also perceptive context. Temporal and syntactic structures in music are reconciled congruently with each other in further cognitive processes and culminate in a whole and complete unit. They are processed in multineuronal pathways, interact hand in hand but also compensate each other. In a musical context, we cannot divide time and rhythm perception from processing superior structures, such as syntax. In this context, Koelsch talks of a “*a multidomain capacity of human cognition”* (Koelsch et al., 2013). According to Fitch, it is also necessary to emphasize that our model includes the processing of rhythmic syntax which should be considered indispensable as “*another important form of musical syntax”* (Fitch, 2013, p.8). It is important to emphasize that in contrast to Grahn's experiment in which all patients were at early stages of the disease (H&Y 1–2), our patients were in advanced stages (H&Y mean 4.3; SD 2.1), and the results in the JND did not show any significant difference compared to controls. Recent findings of Cameron et al. even confirm that PD patients improve their ability to discriminate metric simple rhythms under dopaminergic medication: “*Judging the beat in music does not appear to be affected by PD or by dopaminergic medication*” (Cameron et al., 2016, p. 1). In a beat alignment test, PD patients in an ON state did not perform worse than healthy controls. The authors explain that these results are due to “*different underlying cognitive processes*” (Cameron et al., 2016, p. 6), an explanation which also supports the hypothesis of our study.

**Figure 9:**
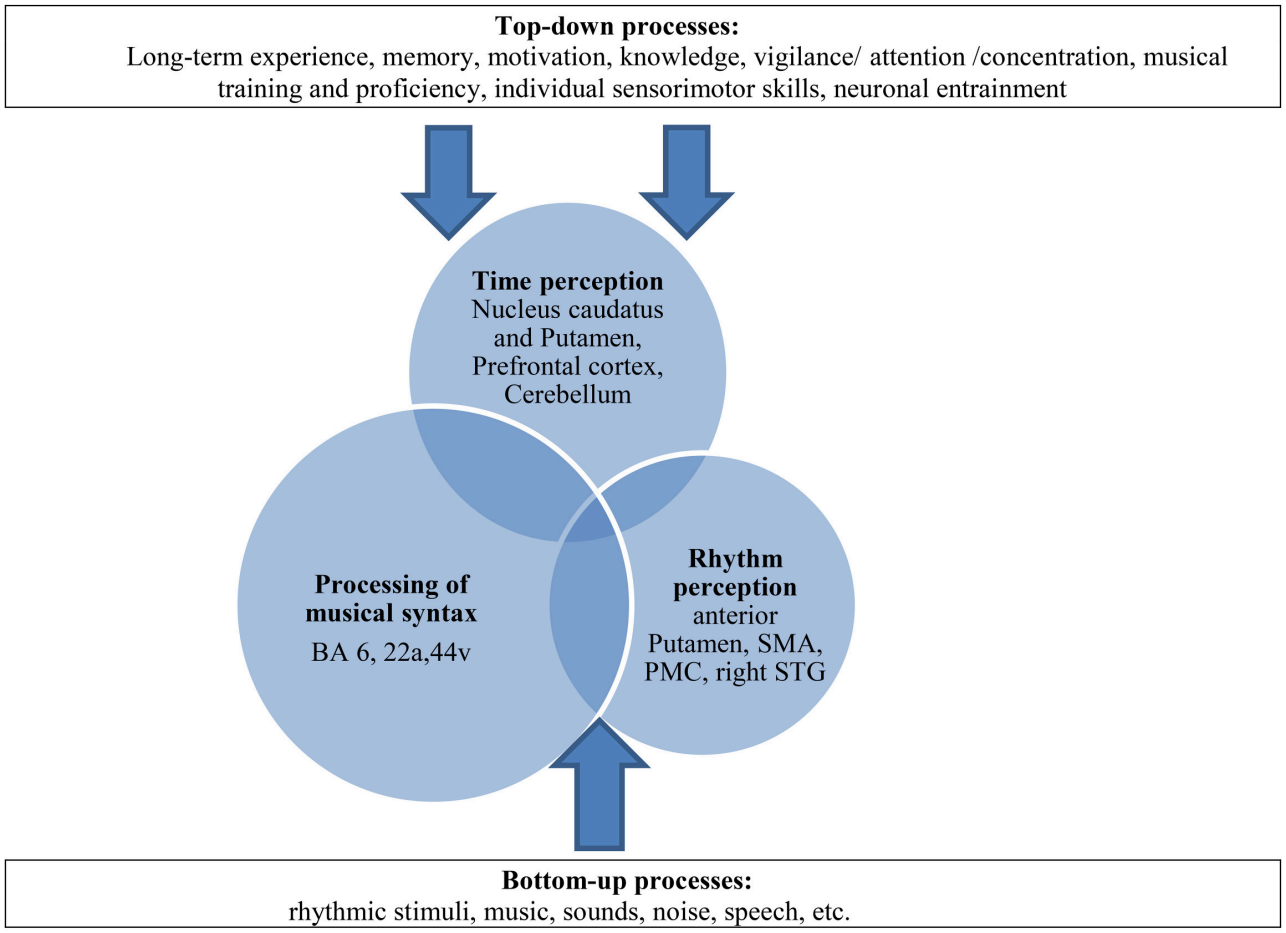
**Multifactorial explanation approach as a *dynamic model of compensation* in patients with PD: paradigm of time-syntax-congruency in music perception as interaction of the three “domains” (overlapping circles) and its modulation with bottom-up-top-down-processes (arrows)**. The processing of musical syntax takes up a greater part with compensatory effects on existing time and rhythm perceptive deficits, shown as larger circles. The processing of musical syntax includes the processing of rhythmic syntax (Fitch, 2013). SMA, supplementary motor area; PMC, premotor cortex; STG, superior temporal gyrus. BA, Brodmann area (for further explanations and literature reference of anatomic correlates of the three domains and top-down-bottom-up-processes please see text).

Furthermore, we assume that bottom-up-top-down processes may play an important role in our paradigm by reinforcing this compensation mechanism. These processes have already been described in the context of processing musical syntax, sensomotoric integration or time processing (Flowers and Robertson, 1995; Engel et al., 2001; Meck, 2005; Koelsch, 2011; Nozaradan et al., 2013) and strengthen the idea that cognitive processes modulate complex neural pathways additionally. The BG which are involved in different functional motoric and sensoric pathways rather operate in these interactions of higher cognitive processes (Kung et al., 2013). Top-down processes, such as long-term experience, memory, motivation, knowledge, attention, musical training but also individual sensorimotor skills or neuronal entrainment (Nozaradan et al., 2013) interact with perceptive bottom-up processes and contribute to this compensation model in a constructive way (see Figure 9).

### Music comprehension is related to a finer resolution in time

The correlation of the JND results with musical expertise was significant: namely, that refined musical expertise coincided with lower JNDs. Although the evaluation of an individual's musical aptitude requires more sophisticated testing than our 12-point-based music score, it turned out to be a sufficient instrument in the context of this study. The present study indicates that individuals with higher musical expertise have more precise discrimination ability for time perception in comparison to subjects with no history of musical training. This is not astonishing, as making music requires auditory, cognitive and sensorimotor skills and therefore improves abilities, such as the perception of time, rhythm or musical syntax. A study with 36 professional musicians and 36 non-musicians confirm the superior perceptive abilities and temporal information processing of musicians (Rammsayer and Altenmüller, 2006; Rammsayer et al., 2011). Although the subjects of our study were all amateurs and not professionals, the significance was only observed between the extreme categories of low and high musical expertise as seen above in Figure 8. This underlines the fact that individuals with formal musical training and current musical activity achieve results similar to those of professionals and perform better than “non-musicians.”

Furthermore, the understanding and extracting of rules constituting musical syntax is a crucial element in playing an instrument or singing. Indeed, the comprehension of these rules affects the individual interpretation of a musical piece and establishes a style of playing music. Therefore, the benefit in temporal and syntactic processing reflected by the JND of “musicians” in comparison to the “non-musicians” can be expected. This fact is in keeping with studies based on differences in anatomical structures (Schlaug, 2006; Woelfle and Grahn, 2013) or electrophysiological activity of musicians and non-musicians (Besson et al., 1994; Boh et al., 2011; Fitzroy and Sanders, 2012; Brattico et al., 2013). Specifically the ERAN, a marker of rule-based syntactic music processing, can be modulated by musical training (Koelsch, 2013, p. 149–150).

## Conclusion

The present study shows that PD patients are impaired in their ability to discriminate temporal deviations in music perception compared to healthy controls. This merely affects the recognition in longer time intervals (>600 ms) and the results can be interpreted as fluctuations of attention and working memory capacity due to the disease. Having presented subjects with clear rhythmic structures and varying temporal Gestalt-parameters, we could indicate that interferences occur with other neuronal networks which can be recruited and utilized as strategies of compensation. Some belong to the processing of temporal (cerebellum) and music perceptive attainments, especially the processing of musical syntax (BA 6, 22, 44). Possible deficits in perception can be compensated for by mechanisms of musical syntax processing because temporal and syntactic structures in music support reconciliation of time-estimation (hypothesis of time-syntax-congruency in music perception) on a global level. Furthermore, top-down-bottom-up processes as multimodal interactions contribute to this compensation mechanism. The stage of the disease does not inevitably correlate with deficits in perception of temporal structures, although as the disease progresses, this compensation model supported by Gestalt principles collapses and can be associated with worse perception attainments. This study also indicates that individuals with a higher musical comprehension and musical training have a better discrimination ability in temporal processing, which becomes especially evident in the temporal lower range of interval (<500 ms).

## Author contributions

DB (doctoral candidate): Planing and execution of the study (doctoral thesis), writing of the paper and correction. EA (consultant, expert): Planning, counseling of the study, proofreading, and correction of the paper. JV (doctoral thesis supervisor): Planning, supervision of the study, proofreading, and correction of the paper.

### Conflict of interest statement

The authors declare that the research was conducted in the absence of any commercial or financial relationships that could be construed as a potential conflict of interest.
