# Supplementary material for: Perception of Time in Music in Patients with Parkinson's Disease–The Processing of Musical Syntax Compensates for Rhythmic Deficits
Source: Front Neurosci. 2017 Feb 23;11:68. doi: 10.3389/fnins.2017.00068 (PMC5322262; doi:10.3389/fnins.2017.00068)
Supplement: Supplementary file 1 [file DataSheet1.docx]

**Appendix**

| **Questions** | **Points** |
| --- | --- |
| Did you learn to play an instrument? | 0 = no  1 = yes |
| Do you like to sing or do you sing in a choir?* | 0 = no  1 = yes |
| Do you currently play an instrument or sing on a regular basis? | 0 = no  1 = yes |
| How many years of musical experience or years of musical activity do you have? | 1-5 years = 1  5- 10 years = 2  > 10 years= 3  > 20 years = 4  > 30 years = 5 |
| Do you listen to classical music? | 0 = no  1 = yes |
| How would you rate your  subjective musical aptitude  (corresponding to the examiner’s  objective impression of musicality  during the experiment)? | 1 = low  2 = medium  3 = high |
|  | **Total: 12** |

**Tab. 1: Assessment of the individual music comprehension in a 12-point music score which was transferred to three different categories of musical comprehension: 1-4p.: low musical comprehension, 5-8p.: average musical comprehension, 9-12p.: high musical comprehension. * The question of liking to sing presumes an sufficient quality of singing and anticipates a minimum of musical aptitude (**[**Mullensiefen, Gingras, Musil, & Stewart, 2014**](#_ENREF_36)**).**
